# Impact of speed and magnitude of weight loss on the development of brain trophic changes in adolescents with anorexia nervosa: a case control study

**DOI:** 10.1186/1824-7288-39-14

**Published:** 2013-02-19

**Authors:** Monica Bomba, Anna Riva, Federica Veggo, Marco Grimaldi, Sabrina Morzenti, Francesca Neri, Renata Nacinovich

**Affiliations:** 1 Department of Child and Adolescent Psychiatry, San Gerardo Hospital, Monza, Italy; 2 Department of Radiology, San Gerardo Hospital, Monza, Italy; 3 Department of Medical Physics, San Gerardo Hospital, Monza, Italy; 4 Clinica di Neuropsichiatria dell’Infanzia e dell’Adolescenza, Ospedale San Gerardo (Nuovo, Scala A, Piano 11), Via Pergolesi, 33, 20900, Monza, Italy

**Keywords:** Adolescent, Anorexia nervosa, Brain, BMI

## Abstract

**Background:**

Anorexia nervosa commonly arises during adolescence and is associated with more than one medical morbidity. Abnormalities in brain structure (defined as “pseudoatrophy”) are common in adolescents with anorexia nervosa; however, their correlations with endocrinological profiles and clinical parameters are still unclear. In particular, no study has described the impact of BMI (body mass index) variations (speed and magnitude of weight loss) on cerebral trophism changes.

**Methods:**

Eleven adolescents with anorexia nervosa and 8 healthy controls underwent cerebral MRI (magnetic resonance imaging) examination to obtain global and partial volumes (gray matter, white matter and cerebrospinal fluid) and clinical evaluation. The Mann-Whitney U test was used to compare partial volumes and clinical variables between cases and controls. The Spearman non-parametric test was performed in order to explore correlations between the variables studied.

**Results:**

The patients diagnosed with AN showed significantly increased cerebrospinal fluid (CSF) volumes and decreased total gray (GM) and white matter (WM) volumes. The degree of weight loss (deltaBMI) correlated inversely with the GM volume; the increase of CSF compartment correlated directly with the rapidity of weight loss (DeltaBMI/disease duration).

**Conclusions:**

This study suggests a correlation between cerebral alterations in AN and the speed and magnitude of weight loss, and outlines its importance for the therapeutic treatment.

## Background

Anorexia nervosa (AN) is a psychiatric illness that commonly arises during adolescence. Among all the psychiatric disorders anorexia has the highest mortality rate and it is associated with severe medical morbidity [1]. Regard to complications, heart abnormalities [2], osteopenia [3], endocrine alterations and brain abnormalities are common.

The typical hypothalamic amenorrhea is associated with low serum levels of gonadotropins and sexual hormones. Plasmatic levels of leptin, a peptide produced by fat store and involved in appetite-regulating mechanisms, are also reduced. GH (growth hormone) levels are often increased accompanied by low levels of IGF-1 (insulin-like growth factor) which suggests an acquired peripheral resistance to GH. An hypercortisolemia is common to almost all women with AN, but not associated to the typical Cushingoid features and often not suppressible after dexamethasone administration. Abnormalities of the thyroid axis are also relevant and characterized by low levels of T_3_ (triiodothyronine), whereas T_4_ (thyroxine) and TSH (thyroid stimulating hormone) are normal or slightly reduced [4,5].

Previous neuroimaging studies on AN showed global gray (GM) and white matter (WM) reduction and an increase in cerebrospinal fluid volumes (CSF) [6,7], while other authors didn’t confirm GM [8,9] or WM decrease [10].

In literature, associations between brain alterations and hormonal profile changes as hypercortisolemia [6,11,12] and low levels of T_3_[12-14] are described.

Weight restoration tends to improve brain abnormalities in AN, but it is still not clear whether reversibility is complete [6,8,10,15,16]. Etiopatogenetic mechanism of cerebral alterations are not still completely explained. First hypothesis that volume reductions are related to neuron death has not been confirmed either by neuro-biochemical [17], histological studies [18] or by improvement of brain alterations with weight restoration [10]. The current hypothesis, summarized in Swayze’s study [8], include: i) decreased serum proteins resulting in decreased colloidal osmotic pressure and a shift of fluid from the intravascular space into the subarachnoid spaces [19]; ii) partial regeneration of damaged neurons and their axons with possible regeneration of myelin [20]; iii) loss of lean body tissue mass [21]; iv) increased urine and serum cortisol levels [22]; v) decreased protein synthesis resulting in loss of dendritic spines, a reduction in the number of synaptic junctions, and delayed synaptogenesis [7].

In literature associations between brain volumes and BMI in AN are known [6], anyway no study explored the impact of speed and magnitude of weight loss on cerebral trophic changes in adolescents with anorexia nervosa. The purpose of our study is to examine this correlation and its implications in the clinical treatment of these young patients.

## Methods

Eleven girls, aged 11–17, who fulfilled the DSM-IV-TR diagnostic criteria for AN were enrolled. They were all new patients of the Eating Disorders Unit of the Department of Child and Adolescent Neuropsychiatry, at the S. Gerardo Hospital in Monza, University of Milano Bicocca (Milan, Italy) from August 2008 to January 2010. Clinical variables such as age, body-mass-index (BMI), age of AN onset, deltaBMI (index of body weight loss which expresses the variation of the BMI between the onset of the disorder and the evaluation), deltaBMI/disease duration (rapidity of BMI variation), the presence of primary/secondary amenorrhea were evaluated by means of a clinical interview. No patient was taking any medication. The Hollingshead 4-factor index was carried out as a measure of the socio-economical status (SES). A normal academic performance was reported for all the girls enrolled. Patients did not have any concomitant medical diseases (except for one girl affected by celiac disease) or psychiatric comorbidity and no prenatal/perinatal/postnatal cerebral suffering was reported.

Eight age-matched girls without psychiatric disorders were also enrolled as controls.

Each subject underwent a high-resolution T1-weighted volume MRI scan Acquisition consists of a set of adjacent axial images with a slice thickness of 1 mm and pixel size 0.94 _ 0.94 mm, using spoiled gradient echo sequence (TR = 25; TE = 4.6 kHz; FOV = 240 cm; matrix 256 _ 256). All MRI data was acquired on the same scanner (1.5 T Achieva Philips) using the same parameters according to strictly standardized procedures. Total cerebral and intracranial volumes (gray matter, white matter, cerebrospinal fluid) were calculated using the FAST and BET extraction tools from the FSL package (http://www.fmrib.ox.ac.uk/fsl/). For each MRI scan a binary mask of cerebral volume was obtained with the BET extraction tool (threshold fixed was 0.5) and then manually outlined.

Parents and participants were told the purpose of the study and a written informed consent to participate was obtained. The research was reviewed and approved by the Institutional Review board.

### Statistics

All continuous variables were expressed as mean ± SD (standard deviation). The Mann-Whitney U test was used to compare partial volumes (GM, WM, CSF), and clinical variables between cases and controls. In the group of adolescents with anorexia nervosa, the Spearman non-parametric test was performed in order to explore correlations between the variables studied. The level of significance was set at p < 0.05. Statistical analysis was performed using the SPSS 19 package.

## Results

The socio-demographic features and BMI of participants with AN and controls are described in Table 1. Subjects with AN presented a mean deltaBMI of 3.9 (S. D. 2.73), a mean time of disease duration of 14.45 (S. D. 10.92) months and a mean deltaBMI/disease duration of 0.70 (S.D. 0.80). Three girls with AN presented primary amenorrhea, eight girls secondary amenorrhea. Nine girls suffered from restrictive and two suffered from purging type of AN.

**Table 1:** Socio-demographic and clinical features of subjects with AN and controls

	**AN group**	**Control group**	**Z**	***p***^***a***^
	**Mean (S.D.)**	**Mean (S.D.)**		
	**n = 11**	**n = 8**		
Age	13.63 (2.77)	13.25 (2.43)	- 0.17	0.87
Hollingshed index	2.73 (1.27)	2.50 (1.69)	- 0.55	0.58
BMI k/m^2^	12.18 (0.87)	19.87 (1.45)	- 3.7	<0.001
DeltaBMI	3.9 (2.73)	-		-
Disease Duration (months)	14.45 (10.92)	-	-	-
DeltaBMI/Disease duration	0.70 (0.80)	-	-	-

^a^ Mann –Whitney *U*-test.

AN = anorexia nervosa.

S.D. = standard deviation.

BMI = body mass index.

DeltaBMI = (Delta body mass index) index of body weight loss which expresses the variation of the BMI between the onset of the disorder and the evaluation.

Differences in WM, GM and CSF volumes, when comparing the two groups (Table 2) were found: participants with AN showed reduced GM (586215 mm^3^, SD = 60592 in the AN group vs 689829 mm^3^, SD = 64033 in the control group; p < 0.005) and WM volumes (465369mm^3^, SD = 44689 in the AN group vs 521839mm^3^, SD = 23645 in the control group; p < 0.02), and higher CSF volumes than controls (315480mm^3^, SD = 55098 in the AN group vs 221033 mm^3^, SD = 24736 in the control group; p < 0.002).

**Table 2:** Comparison of volumetric measures between participants with AN and controls

	**AN group**	**Control group**	***p***^***a***^
	**Mean (S.D.)**	**Mean (S.D.)**	
Gray matter mm^3^	586215 (60592)	689829 (64033)	<0.005
White matter mm^3^	465369 (44689)	521839 (23645)	<0.02
CSF mm^3^	315480 (55098)	221033 (24736)	<0.002

^a^ Mann –Whitney *U*-test.

S.D. = standard deviation.

AN = anorexia nervosa.

CSF = cerebrospinal fluid.

The DeltaBMI was inversely correlated with the fraction of GM volume, which means that girls with AN with the greatest weight loss between the onset of the disease and the time at diagnosis had the greatest reduction of GM volume. Delta BMI also directly correlated with the enlargement of CSF volume. Moreover, a direct correlation was also observed between the CSF compartment volume and the fraction DeltaBMI/disease duration (Table 3).

**Table 3:** Correlations between Volumetric and Clinical variables in the participants with AN

**Clinical variable**	**Volumetric/clinical variable**	**Spearman**	***p***^**a**^
DeltaBMI	Gray matter	−0.724	<0.02
DeltaBMI	CSF	0.631	<0.04
DeltaBMI/Disease duration	CSF	0.618	<0.05

^a^ Spearman non-parametric test.

DeltaBMI = index of body weight loss which expresses the variation of the BMI between the onset of the disorder and the evaluation.

DeltaBMI/disease duration (rapidity of BMI variation).

CSF = cerebrospinal fluid.

## Discussion

To our knowledge, this is the first study conducted on a sample of young adolescents with AN in which correlations between cerebral volumes and speed and magnitude of weight loss are explored. A negative correlation between deltaBMI and the fraction of gray matter volume and a positive correlation between deltaBMI and the enlargement of CSF volume were observed. To the contrary, no correlations between cerebral volume parameters and BMI value were found [10], which may suggest that the degree of weight loss, indicated by deltaBMI, and not the low body weight itself or a longer illness duration might be responsible for the development of a brain pseudoatrophy. A remarkable weight loss might determine a failure in metabolic compensatory mechanisms contributing to cerebral volume alterations. Furthermore a significant correlation was also observed between CSF volume and DeltaBMI/disease duration which could suggest not only the importance of the impact of the weight loss degree but also of the timetable in which this occurs.

Girls with AN displayed significant reduced GM and WM volumes and increased CSF volumes, confirming data from the literature [6]. Differently, girls with AN with a higher BMI than that of our sample showed decreased GM and widened CSF volume, without the alteration of WM volumes [10]. In adult patients with AN, a widening of cerebral sulci, enlarged lateral and third ventricle and a decrease of the WM volume were described [8]. These changes were attributed to lipolysis of the lipids that form the bulk of myelin in both white and gray matter and the reduced levels of IGF-1 have been considered responsible for it. Moreover, the reduction of the brain tissue mass would especially interest the white matter due to its greater proportion of myelin [8].

Our study results outline the importance of an early diagnosis of the illness in order to limit also cerebral alterations. In fact, a precocious intervention and screening programs might be necessary in order to prevent major cerebral alterations of GM, WM and CSF. Concerning this problem, our sample of anorexic adolescents get the first access to our specialized unit with a mean disease duration of more than one year. More awareness is needed among pediatricians and neuropsychiatrists so that adolescents at risk can be promptly recommended a consult with a specialist. The main limitation of the present work was sample size that should be increased in future studies. However, our sample is comparable with other published studies on adolescents with AN. Furthermore our intent was to investigate a group of patients homogeneous for age, type of anorexia and illness severity (all patients had a BMI under 14).

## Conclusions

Anorexia nervosa is a psychiatric illness with a high prevalence and origin in adolescence.

In adolescents with AN, the speed and magnitude of weight loss, and not only BMI, represent a possible indicator of disease severity. If confirmed by other studies, our results could provide additional support concerning the benefits of paediatricians’ and neuropsichiatrists’ increased awareness, early detection and treatment of patients with anorexia nervosa.

## Abbreviations

BMI: Body mass index; MRI: Magnetic resonance; CSF: Cerebrospinal fluid; GM: Gray matter; WM: White matter; DeltaBMI: Delta body mass index; AN: Anorexia nervosa; GH: Growth hormone; IGF-1: Insuline-like growth factor; T_3_: Triiodothyronine; T_4_: Thyroxine; TSH: Thyroid stimulating hormone; DSM-IV-TR: Diagnostic and statistical manual of mental disorders, Fourth Edition, Text Revision; SES: Socio-economical status; SD: Standard deviation; SPSS: Statistical Package for Social Science.

## Competing interests

Authors declare that they have no competing interests.

## Authors’ contributions

MB and RN planned the study. MB, AR, VF and RN conducted patients' clinical evaluation. MG and SM conducted radiological evaluation. MB and SM conducted statistical analysis. All authors contributed to draft the manuscript, which they read and approved in the final version.
